# Supplementary material for: Bacterial Community Shifts Driven by Nitrogen Pollution in River Sediments of a Highly Urbanized City
Source: Int J Environ Res Public Health. 2019 Oct 9;16(20):3794. doi: 10.3390/ijerph16203794 (PMC6843462; doi:10.3390/ijerph16203794)
Supplement: Supplementary file 1 [file ijerph-16-03794-s001.pdf]

Supplementary Material

Bacterial community shifts driven by nitrogen pollution in river sediments of a highly urbanized city

Xianbiao Lin[1,3,4], Dengzhou Gao[3], Kaijun Lu[5], Xiaofei Li[2,3*]

[1]Laboratory of Microbial Ecology and Matter Cycles, School of Marine Sciences, Sun Yat-Sen University, Zhuhai 519082, China

[2]Key Laboratory for Humid Subtropical Eco-geographical Processes of the Ministry of Education & School of Geographical Sciences, Fujian Normal University, Fuzhou, 350007, China

[3]School of Geographic Sciences, East China Normal University, Shanghai, 200241, China

[4]Southern Laboratory of Ocean Science and Engineering (Guangdong, Zhuhai), Zhuhai, 519000, China

[5]The University of Texas at Austin Marine Science Institute, 750 Channel View Drive, Port Aransas, Texas 78373, USA

***Corresponding author**：Phone: 86-591-83465214; fax: 86-591-83465397, E-mail address: lixiaofei198702@126.com

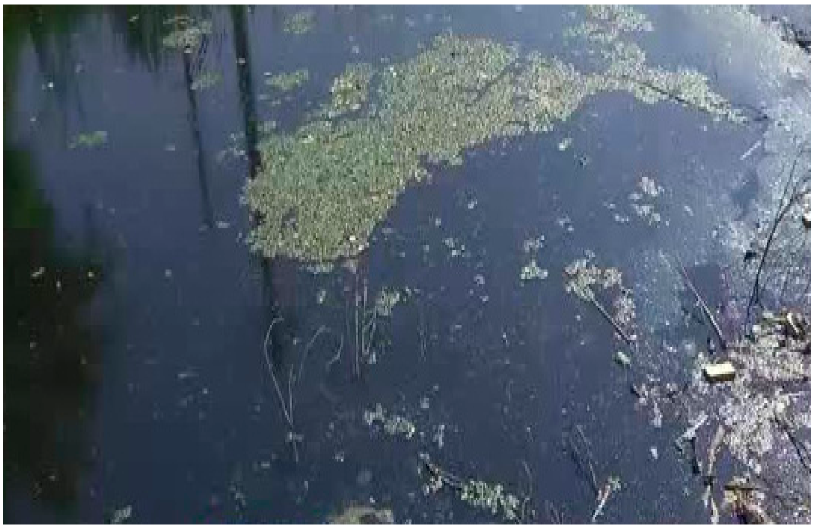

Fig. S1. The landscape of a river heavily polluted by anthropogenic wastewater discharge in the study area

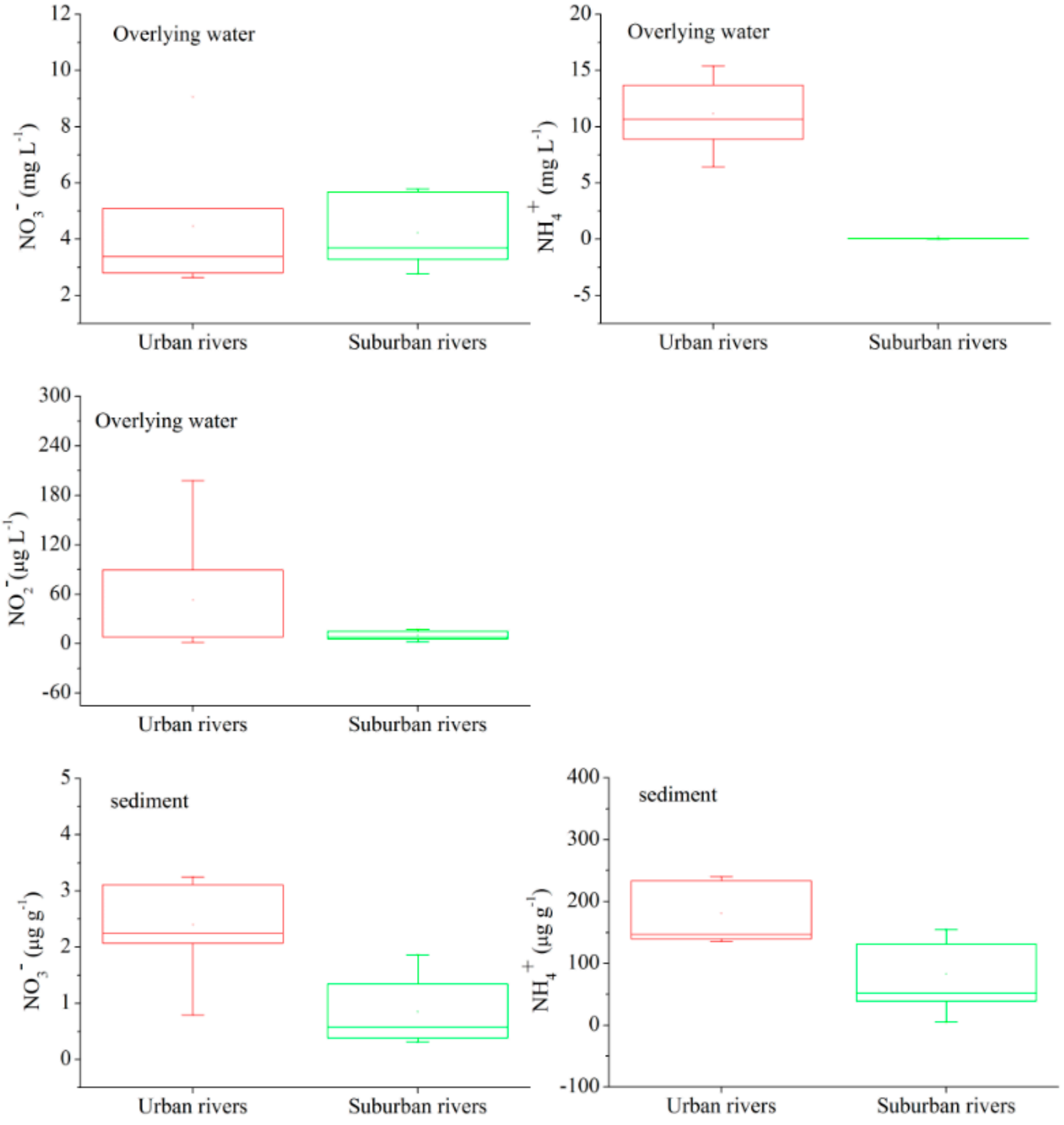

Fig. S2. Concentrations of inorganic nitrogen forms in urban and suburban rivers

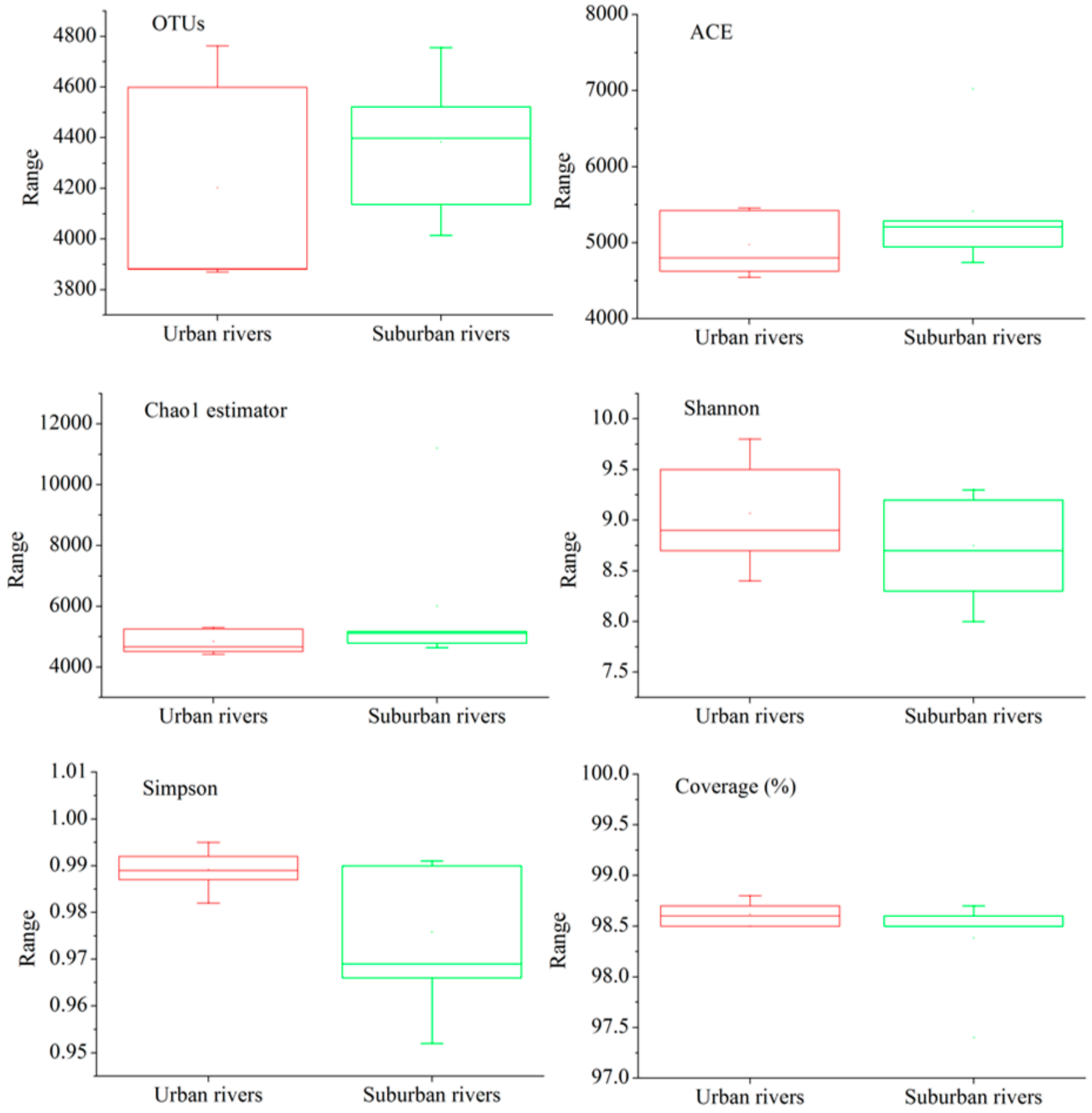

Fig. S3. Diversity indices of bacterial community in urban and suburban rivers

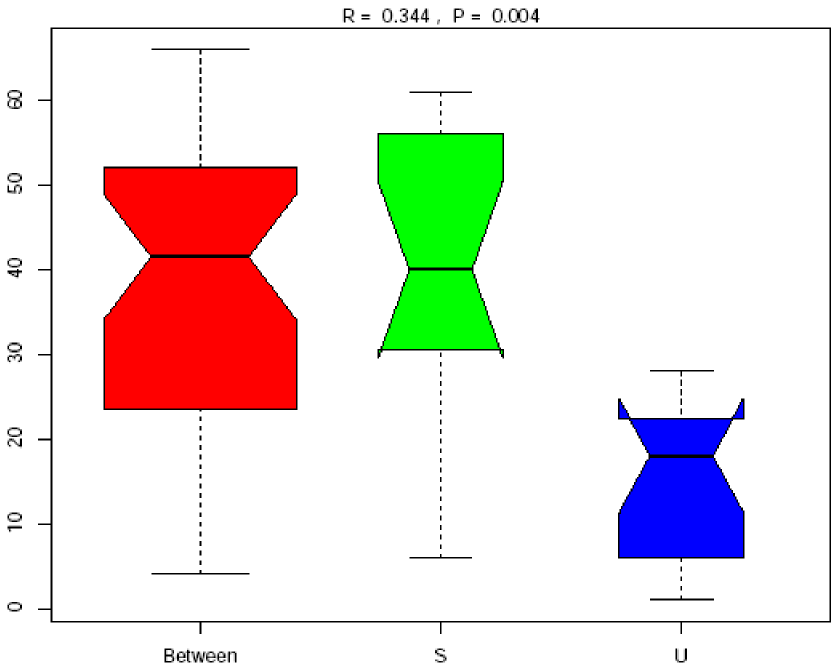

Fig. S4. Analysis of similarity about the bacterial community.

Table S1. Relative abundance (%) of top 36 abundant families in the samples. *P*_value of ANOVA analysis indicated significant difference of bacterial abundance between grouped urban and suburban rivers.

Phylum	Family	Urban rivers		Suburban rivers		*P*_value
		range	mean	range	mean	
Acidobacteria	*Acidobacteriaceae_.Subgroup_1*	0.02–1.42	0.37	0.02–0.56	0.13	0.34
	Gemmatimonadaceae	0.31–1.96	0.64	0.32–1.55	0.69	0.89
Bacteroidetes	*WCHB1-69*	0.29–1.51	0.68	0.15–0.33	0.24	**0.04***
	Chitinophagaceae	0.15–0.43	0.27	0.05–0.34	0.44	0.51
	Flavobacteriaceae	0.52–2.78	1.28	0.65–7.08	2.09	0.46
Chloroflexi	*Anaerolineaceae*	1.98–5.67	3.4	2.34–4.38	3.18	0.75
Cyanobacteria	*unidentified_Chloroplast*	0.04–1.29	0.54	0.03–0.12	0.18	0.15
Firmicutes	*Planococcaceae*	1.03–1.21	1.14	1.91–8.85	4.08	**0.04***
Gemmatimonadetes	*Gemmatimonadaceae*	0.31–1.96	0.64	0.32–1.55	0.69	0.89
Proteobacteria	*Syntrophaceae*	1.52–2.98	1.92	0.85–1.76	1.27	**0.026***
	Bacillaceae	0.17–0.29	0.21	0.23–21.32	6.57	0.14
	Pseudomonadaceae	0.73–2.70	1.62	0.77–2.01	1.31	0.38
	Comamonadaceae	2.60–6.08	3.42	2.33–5.43	2.99	0.57
	Helicobacteraceae	0.40–2.27	0.94	0.16–2.80	1.19	0.63
	Rhodocyclaceae	6.26–12.96	9.83	4.17–8.65	5.74	**0.005****
	Geobacteraceae	2.08–3.96	3.03	1.33–2.13	1.75	**0.0018****
	M20-Pitesti	0.50–1.40	0.71	0.27–0.69	0.43	0.10
	Acetobacteraceae	0.19–1.29	0.59	0.18–0.48	0.26	0.17
	Gallionellaceae	2.10–4.74	3.04	1.39–4.27	2.66	0.54
	Clostridiaceae_1	0.48–0.75	0.58	0.58–4.03	1.64	0.07
	Desulfobacteraceae	0.76–1.89	1.15	0.50–1.09	0.84	0.17
	Enterobacteriaceae	0.51–11.51	3.42	0.50–17.70	4.39	0.77

	Moraxellaceae	0.70–1.72	1.00	0.63–3.07	1.82	0.056
	unidentified_Xanthomonadales	1.55–2.46	1.88	0.73–1.37	0.99	**0.0004****
	Hydrogenophilaceae	1.68–3.73	2.60	1.13–3.53	2.26	0.46
	Nitrosomonadaceae	0.59–1.95	0.97	0.53–1.26	0.93	0.90
	Oxalobacteraceae	0.68–1.29	0.87	0.68–9.68	2.66	0.24
	Alcaligenaceae	0.74–1.16	0.94	0.45–1.09	0.78	0.21
	Crenotrichaceae	0.42–1.17	0.68	0.28–0.74	0.44	0.12
	Desulfobulbaceae	1.31–2.16	1.65	1.22–2.10	1.53	0.61
	Xanthomonadaceae	1.68–3.31	2.61	1.60–3.30	2.38	0.66
	Burkholderiaceae	0.10–3.80	0.88	0.08–0.45	0.16	0.25
	Spongiibacteraceae	1.03–1.52	1.33	0.62–1.16	0.82	**0.0018****
	Sphingomonadaceae	0.40–0.96	0.65	0.41–2.09	0.75	0.73
Spirochaetes	*Spirochaetaceae*	0.81–1.89	1.12	0.54–1.04	0.85	0.17
Verrucomicrobia	*Verrucomicrobiaceae*	0.18–1.47	0.47	0.16–0.38	0.24	0.30

*$P < 0.05$, **$P < 0.01$

Table S2. Relationships between properties and relative abundances of phylum bacterial communities.

	Proteobacteria	*Firmicutes*	*Bacteroidetes*	*Chloroflexi*	*Actinobacteria*	*Acidobacteria*	*Verrucomicrobia*	*Spirochaetes*	*Gemmatimonadetes*	*Nitrospirae*
pH [a]	-0.63*	0.66*	-0.17	0.20	0.21	0.25	-0.12	-0.35	0.18	0.61*
DO [a]	-0.25	0.21	0.26	-0.11	0.61*	0.05	-0.57	-0.76*	0.63*	0.47
DOC [a]	0.53	-0.42	0.26	-0.34	-0.21	-0.41	0.10	-0.24	-0.20	-0.61*
NO_3^- [a]	-0.11	0.10	0.11	-0.24	0.19	0.10	-0.13	-0.01	0.08	0.05
NH_4^+ [a]	0.54	-0.63*	0.08	-0.06	-0.13	-0.11	0.39	0.25	0.04	-0.65*
NO_2^- [a]	-0.08	-0.19	0.43	0.24	0.15	0.17	0.74**	0.09	0.10	-0.28
Fe(II) [b]	0.60*	-0.26	-0.35	-0.39	-0.70*	-0.36	-0.01	0.02	-0.43	-0.48
Sulfide [b]	-0.57	0.53	0.19	-0.04	0.41	0.14	-0.31	-0.50	0.37	0.59*
TOC [b]	-0.36	0.32	-0.56	0.43	0.01	0.39	0.45	0.23	0.32	-0.04
TN [b]	-0.22	-0.12	-0.02	-0.52	0.20	0.56	0.82**	0.43	0.22	0.03
C/N [b]	-0.08	0.40	-0.37	-0.16	-0.30	-0.31	-0.39	-0.15	-0.09	-0.14
NO_3^- [b]	0.48	-0.59*	0.31	-0.21	0.05	-0.10	0.19	0.07	0.20	-0.41
NH_4^+ [b]	0.54	-0.30	-0.13	-0.54	-0.60*	-0.45	0.27	0.33	-0.34	-0.87**

Note: [a] indicated properties of overlying water, [b] indicated properties of sediment. *$P < 0.05$ and **$P < 0.01$ indicated significant correlation.

Table S3. Correlations among geochemical properties in overlying water and sediments.

	pH [a]	DO [a]	NO_3^- [a]	NH_4^+ [a]	NO_2^- [a]	Fe(II) [b]	Sulfide [b]	TOC [b]	TN [b]	C/N [b]	NO_3^- [b]	NH_4^+ [b]
pH [a]		0.24	-0.18	-0.78**	-0.15	-0.03	0.79**	0.15	-0.11	0.30	-0.79**	-0.66*
DO [a]			0.22	-0.17	-0.29	-0.36	0.68*	-0.25	-0.46	0.12	0.11	-0.29
NO_3^- [a]				-0.17	-0.32	-0.24	0.03	0.04	-0.02	-0.10	0.18	0.26
NH_4^+ [a]					0.39	-0.02	-0.58*	-0.06	0.19	-0.31	0.70*	0.62*
NO_2^- [a]						-0.20	-0.25	0.16	0.65*	-0.44	0.27	0.03
Fe(II) [b]							-0.26	0.05	-0.28	0.40	-0.12	0.40
Sulfide [b]								-0.08	-0.34	0.29	-0.41	-0.52
TOC [b]									0.53	0.34	0.03	0.19
TN [b]										-0.58*	0.25	-0.01
C/N [b]											-0.30	0.13
NO_3^- [b]												0.56*
NH_4^+ [b]												

Note: [a] indicated properties of overlying water, [b] indicated properties of sediment. $^*P < 0.05$ and $^{**}P < 0.01$ indicated significant correlation.
